# Some Observations on Fifty Cases of Whooping Cough

**Published:** 1878-08

**Authors:** C. F. Swan

**Affiliations:** South Chicago, Ill.


### SOME OBSERVATIONS ON FIFTY CASES OF WHOOPING COUGH.

By C. F. Swan, M. D., of South Chicago, Ill.

During the spring months of the current year, the suburban town of South Chicago, where I am engaged in practice, was visited by an epidemic of whooping cough. It is well known that the town is situated at the junction of Lake Michigan and the Calumet river, in the neighborhood of submerged soil and swamp lands, and this circumstance, taken in connection with the unusally cold and rainy months of the spring of 1878, may have contributed somewhat to the severity and complications of the cases recorded below.

Having had the opportunity of observing fifty patients affected with this disease in my own practice, I have concluded to make some observations founded upon the therapeutical results obtained by the treatment pursued, though the cases themselves were not, as far as I can determine, unique either in symptoms or course. Unfortunately, this conclusion was reached only after the disappearance of the disease, and, as a result, I have to depend upon the notes taken from my case-book at the time of attending each patient, rather than from details carefully collected with a view to publication. This will explain the very brief and defective record appended to many of the reports.

The first ten patients were treated in the manner detailed below, and without any attempt at establishing the efficiency of any special remedy in shortening the natural period of the disease, or in abating the frequency and severity of the paroxysms. The different medicaments used were: quinine, cod liver oil, tincture of gelseminum, and anodynes in general, with the local inhalation, in two cases, of carbolized steam spray.

After these ten patients had been treated, I determined to make trial of a formula which was suggested to me by Dr. James Nevins Hyde, of Chicago, in a conversation when the subject of the therapeutics of pertussis was under discussion. The results of this treatment in the forty cases subsequently seen by me, can best be studied by a survey of the records themselves. I may, however, here anticipate somewhat, by saying that in some instances the results were as brilliant as they were gratifying, and that in some others the remedy employed seemed to have no effect whatever, the relative proportion of these cases will appear subsequently. I merely remark that it would seem the best results were obtained when the treatment was begun before any serious complications occurred, though in cases of pneumonia, with constant irritation of the glottis and a teasing desire to cough, marked relief was obtained.

The simple formula* used was first suggested in Germany. It is a solution of quinine and tannic acid, 4 Gms. of the former to 1 Gm. of the latter, in water, syrup, licorice or any agreeable elixir, the dose to be proportioned to the age of the child. Dissolved in water in the proportions given, a milky precipitate is formed by the insoluble tannate of quinia, much resembling an emulsion in appearance, and requiring that the bottle be shaken before the administration of each dose. A point worthy of special note, is that the remedies named, in order to produce the effect, should be used in solution. There is thus produced upon the fauces and epiglottis a marked local effect, due to the intensely bitter taste of the quinia, a local effect which unquestionably contributes to the value of the medication. As a consequence, many children utterly refuse to swallow a second dose; and one who is familiar with the symptoms of the disease, need not be told that persistent attempts to induce such children to take medicine of any kind, usually precipitate and aggravate a paroxysm. Some children, however, are not only readily induced to swallow the solution, but, experiencing the value of the medication, present themselves voluntarily, at proper intervals, for the purpose of taking their medicine, a few even seeming to enjoy it. It is, therefore, to the local action of quinine upon the fauces and glottis as well as to its anti-spasmodic virtues, that the results in favorable cases are to be attributed.

---

* [ This treatment was first advised by Prof. C. Binz, of Bonn, Cf. *Jahrbuch für Kinderheilk.* I p, 233, Leipzig; 1868; also *Obstet. Journ.*, May, 1870, Vol. III.—Ed.]

| Number. | Age, Years. | Stage of Disease when First Observed. | Grade and No. of Paroxysms in 24 Hours. | Treatment. | Duration of Disease. | Deaths. | Complications, Etc. |
|---|---|---|---|---|---|---|---|
| 1 | 4 | ...... | ........... | Expectant. | 5 w. | .. | [conj'l hæm. |
| 2 | 68 | B. 1 st. | 40 | Carbol. Spray | 10 w. | .. | Vomiting, gastric catarrh, |
| 3 | ¼ | ...... | Very sev're | Expectant. | 16 w. | .. | Umbilical hernia. |
| 4 | 7 | ...... | 80 | An. Car. Spy. | ..... | .. | Vomiting, emaciation. |
| 5 | 4 | ...... | Very mild. | None. | 3 w. | | { Catarrhal symp., reten- |
| 6 | 1½ | ...... | Very sev're | Qu.cod.liv.oil | 8 w. | .. | tion of urine, struma, |
| 7 | .... | ...... | Mild. | Anodynes. | | | syncope, nasal and buc- |
| 8 | .... | ...... | Mild. | Anodynes. | | | cal hæmorrhage. |
| 9 | 9 | ...... | 8. | Gel.tr.30c.c.q.iv.h | .... | .. | Slight fever. |
| 10 | 7 | ...... | 8. | " " | ..... | . | Slight fever. |
| 11 | 2 | B. 3 w. | Very sev're | Qui. & Tann. | 3 d. | .. | Pneumonia. |
| 12 | 1/12 | ...... | ........... | ............. | 1 d. | 1 | Spasm of glottis. |
| 13 | 2 | 2 w. | Const. cou. | Qui. & Tann. | 2 d. | .. | Fever slight; med. rel'ed. |
| 14 | 1/6 | 2 st. | Very sev're | Qui. & Tann. | 3 w. | .. | Medicine valueless. |
| 15 | 2 | 2 w. | 12. | Qui. & Tann. | 10 w. | .. | Headache; med. valueless |
| 16 | 7 | ...... | Mild. | Expectant. | ..... | .. | Second attack. |
| 17 | 2 | ...... | ........... | ............. | 16 w. | 1 | Marasmus. |
| 18 | 10 | 10 w. | ........... | Expectant. | 14 w. | .. | Pneumonia. |
| 19 | 10 | 2 w. | Distinct. | Qui. & Tann. | 2 d. | .. | Disease aborted. |
| 20 | 14 | 2 w. | Distinct. | Qui. & Tann. | 2 d. | .. | Disease aborted. |
| 21 | 6 | 2 w. | Distinct. | Qui. & Tann. | 2 d. | .. | Disease aborted. |
| 22 | 3 | 2 st. | 24. | Qui. & Tann. | 2 d. | .. | Disease aborted. |
| 23 | 1 | ...... | ........... | Med. refused. | | | |
| 24 | 8 | ...... | Mild. | Qui. & Tann. | 3 w. | .. | Second attack. |
| 25 | 6 | ...... | Mild. | None. | | | [valueless. |
| 26 | 3 | ...... | Severe. | Qui. & Tann. | 6 w. | . | Nasal & bucc. hæm. Med. |
| 27 | 4 | B. 2 st. | ........... | Qui. & Tann. | 4 d. | .. | Disease aborted. |
| 28 | 7 | ...... | Mild. | Qui. & Tann. | | | |
| 29 | 3 | 2 st. | Parox. freq. | Qui. & Tann. | .... | .. | Anodynes at night. |
| 30 | 5 | ...... | No | record. | | | |
| 31 | .... | ...... | Mild. } | | | | |
| 32 | .... | ...... | Mild. } | Refused | | | |
| 33 | .... | ...... | Mild. } | Medicine. | | | |
| 34 | .... | ...... | Mild. } | | | | |
| 35 | 2 | 4 w. | Severe. | Qui. & Tann. | 3 d. | .. | Capillary bronchitis. |
| 36 | 1½ | ...... | Mild. | Qui. & Tann. | | | |
| 37 | 4 | ...... | Mild. | Qui. & Tann. | ..... | .. | [of urine; 3d week measles. |
| 38 | 6 | ...... | 60. | Qui. & Tann. | 7 d. | .. | Struma, nasal hæm., syn., reten. |
| 39 | 2 | 10 w. | 48. | Qui. & Tann. | 10 d. | .. | Hæm. nas. & bucc., incont. of urine |
| 40 | 13 | 3 w. | Severe. | Qui. & Tann. | 14 d. | .. | & fæc. dur. Par. Capil'ry bronc'ts. |
| 41 | 6 | ...... | Mild. | Qui. & Tann. | 4 w. | | Croupous pneumonia. |
| 42 | 1 | B. 2 st. | ......... | Qui. & Tann. | 2 d. | .. | Vomiting with Paroxysms. |
| 43 | 6 | B. 2 st. | 24. | Qui. & Tann. | mrkd | re | lief. Emaciation. |
| 44 | 4 | ...... | Mild. | Refused Med. | | | |
| 45 | 32 | 3 w. | Severe. | Qui. & Tann. | mrkd | re | lief. Pneumonia. |
| 46 | 5 | 7 w. | Severe. | Qui. & Tann. | 7 d. | .. | Pneumonia. |
| 47 | 10 | ...... | 50. | Qui. & Tann. | 10 d. | .. | Syncope, cap. bron, delir. |
| 48 | 3 | ...... | Mild. | Qui. & Tann. | no rlf | .. | from treatment. |
| 49 | 2 | 1 w. | Marked 24. | Qui. & Tann. | 2 d. | .. | Disease aborted. |
| 50 | 6 | ...... | ........... | Qui. & Tann. | no rlf | .. | from treatment. |

The following cases may be given in somewhat fuller detail:

Case XI. Millie N., æt. 2 years. I saw her first at the beginning of the third week on the fourth day of the second stage. Pulse, 160. Temperature, 40° C. Respiration, 60. Had eaten nothing for 48 hours. Dullness and absence of vesicular murmur over right lung. Stupid. Quin. sulph. 2 grams, tannic acid .50 Gm., syrup. glycyrrh. co. 250 C. C. S. Teaspoonful every four hours, also tincture of aconite root .03 C. C., mustard bath, and cold to head.

2nd day. Pulse, 140; temperature, 39.5° C. Had slept well on the previous night.

3d day. Temperature, 38° C.; pulse, 102. Perspired during the night and only coughed once.

4th day. Countenance, bright; pulse, 100; temperature, normal; appetite, good. Only one paroxysm in twenty-four hours; expectorated without any effort, a frothy mucus, tinged with blood. Convalescence from this date.

Case XVIII. H. J., æt. 10 years. I first saw her on the tenth week of the disease. Pulse, 140; temperature, 40.5° C.; respiration, 45. She had great pain in the right lung with expectoration of a large amount of rusty mucus. Finally, the entire right lung became solidified. Convalescence was slow and accompanied by the production of albuminuria and anasarca. Duration of disease, fourteen weeks.

Case XXVII. Hulda B., æt. 10 years. Twelfth week of tussis convulsiva. 'Caught cold' and had chill, followed by fever and acute mania. I found the patient talking incoherently, with temperature of 41.6° C.; pulse, 140; respiration, 72, and perfectly deaf. Paroxysms every half hour, lasting five minutes, and frequently ending in syncope. Mucous and sonorous râles very loud over the whole chest. Ordered: quinine sulphate .12 Gm., tannic acid .033 Gm., alternately with tincture of gelseminum 0.333 C. C., every four hours and frequent sponging of the whole surface. Bromide of potassium .60 Gm. at night, which secured rest. Convalescent in ten days, hearing regained, and cure complete.

Case XXXV. Mary H., æt. 2 years. I saw her first in the fourth week of the disease. The paroxysms were very frequent with loud râles, audible over the entire surface of the chest. ulse 140; temperature 40° C.; appetite poor. I gave sulphate of quinine .100 Gm., tannic acid .016 Gm. every four hours with good results. On the third day, the cough had nearly disappeared, and the paroxysms were much less severe and frequent. She expectorated freely a tenacious yellowish mucus. After this date I lost sight of the patient.

Case XLV. Mrs. McV., æt. 32 years. She had been coughing three weeks when I first saw her. Pulse 140; temperature 40.5° C. Dyspnœa and paroxysms very frequent and painful. Dullness over lower lobe of right lung; rusty sputa. I gave her quinine sulphate .60 Gm. at *bed time.* Tinct. of aconite root 30 C. C. every four hours. Morph. .016 Gm. hypodermically. On the next morning, pulse 100; temperature 37° C.; paroxysms less frequent and painful. Ordered quiniæ sulph. .18 Gm., acid tannic .06 Gm. every four hours, with good effect.

Case XLVI. Oscar M., aet. 5 years. In the seventh week of tussis convulsiva, had not lain down for three nights and days, but sat in a rocking chair. No appetite; pulse 140; temperature, 40.3° C.; respirations hurried, about 40 per minute. Photophobia and mental irritability; dullness over lower lobe of right lung, and absence of vesicular murmur; urine scanty; bowels costive; tongue furred. Ordered quiniæ sulphas 0.12 Gm., acid tannic, .033 Gm., every four hours, with tincture of gelseminum, .30 C. C. Paroxysms every hour, and oftener, if irritated. Improvement marked. Seventh day, convalescent. No cough.

Remarks: Excluding the two cases occurring in adults, aged respectively 68 and 32 years, the average age of the 42 children whose ages are recorded, was 4.7 years. Of the 22 cases, in which a record was made of the period of the disease in which the patients were first submitted to treatment, 6 were seen at the beginning of the second stage, 6 in the second week, 2 at the beginning of the first stage, 2 in the third week, 2 in the 10th week and 1 in the fourth week. Of the 30 cases in which a memorandum was made relative to the severity of the disease, 21 were registered as mild, the symptoms being well marked, 5 as severe and 4 as very severe. The average number of paroxysms in 24 hours, in the case of 10 children, was 32.8. Five cases were complicated with pneumonia, 4 with nasal hemorrhage, 3 with buccal hæmorrhage, 3 with capillary bronchitis, 3 with fever, 3 with vomiting at the time of the paroxysm, 2 with retention of urine, and 1 each with conjunctival hæmorrhage, umbilical hernia, spasm of the glottis, headache, marasmus, struma, incontinence of urine and fæces at the time of the paroxysm, and delirium.

As regards the results of the treatment—the most interesting point in this connection, the table shows the following results: Of the forty cases visited after the quinine medication was tried, three were so mild as to require no treatment; no record is given in six cases; and six children utterly refused the medicine in consequence of its bitter taste. Two died, one of spasm of the glottis, a baby one month old whom I saw but once; the other of marasmus, after lingering four months. Deducting, then, 17 cases from the 40, we find 23 left. Of these 23, 8 patients received no benefit whatever from the quinine and tannin, or at least the duration of the disease did not seem to be shortened in any degree by the treatment. In two cases "marked relief" was afforded, the duration of the disease not being noted.

*But in the case of the fifteen patients not heretofore considered, the disease was completely aborted in an average period of* 3.8 days, three of the cases being complicated by pneumonia and two by capillary bronchitis. Due allowance being made for the limited number of observations, the results of which are given above, I think it not unreasonable to conclude that the use of a solution of quinine and tannin internally in whooping cough, may be employed with advantage in many cases; and that in nearly 60 per cent. of the little patients who can be persuaded to swallow it, the disease will be completely arrested in about four days.

